# Safety and Efficacy of Anti-PD-1 Monoclonal Antibodies in Patients With Relapsed or Refractory Lymphoma: A Meta-Analysis of Prospective Clinic Trails

**DOI:** 10.3389/fphar.2019.00387

**Published:** 2019-05-01

**Authors:** Hui Zhou, Xiaoyan Fu, Qian Li, Ting Niu

**Affiliations:** Department of Hematology and Research Laboratory of Hematology, West China Hospital, Sichuan University, Chengdu, China

**Keywords:** anti–PD-1 monoclonal antibodies, nivolumab, pembrolizumab, relapsed or refractory lymphoma, safety, efficacy

## Abstract

**Background:** Immune checkpoint inhibition therapy with monoclonal antibody against programmed cell death protein 1 (PD-1), including nivolumab and pembrolizumab, has demonstrated powerful clinical efficacy in the treatment of advanced cancers. However, there is no evidence-based systematic review on the safety and efficacy of anti-PD-1 antibody in treating lymphoma.

**Methods:** To evaluate the safety and efficacy of nivolumab/pembrolizumab, we analyzed clinical trials from PUBMED, EMBASE, and The Cochrane Library. For safety analysis, the incidence and exhibition of any grade and grade ≥3 adverse events (AEs) were evaluated. Overall response rate (ORR), 6-month progression-free survival (PFS) and 6-month overall survival (OS) were calculated for efficacy analysis.

**Results:** Overall ten studies and 718 patients (114 non-Hodgkin lymphomas, 604 Hodgkin lymphomas) were enrolled, including 4 phase I studies and 6 phase II studies. The pooled incidences of any grade and grade ≥3 adverse events (AEs) were 74 and 24%, respectively. Drug-related deaths occurred in two patients. The most common any grade AEs were fatigue (14.91%), rash (14.8%), hypothyroidism (13.77%), platelet count decreased (13.54%), pyrexia (13%). The most common grade ≥3 AEs were neutropenia (4.79%), pneumonitis (3.58%), rash (3.38%), and leukopenia (3.31%). Fatigue (*p* = 0.0072) and rash (*p* = 0.0078) in any grade AEs were less observed in patients treated with pembrolizumab than nivolumab. The pooled ORR, PFS rate and OS rate were 58, 73, and 96%, respectively. The ORR in patients with Hodgkin lymphomas (HL) was higher than patients with non-Hodgkin lymphomas (NHL) (69.08 vs. 30.77%, *p* < 0.0001). However, there was no significant difference of efficacy between nivolumab and pembrolizumab.

**Conclusions:** Nivolumab and pembrolizumab have promising outcomes with tolerable AEs and drug-related deaths in patients with relapsed or refractory lymphoma. Pembrolizumab caused less any grade AEs like fatigue and rash than nivolumab. Patients with HL got better response than NHL.

## Introduction

Programmed cell death protein 1 (PD-1) is an immune checkpoint receptor mainly expressed on activated T cells, natural killer cells, and B cells (Ishida et al., 1992). The PD-L1 and PD-L2 are its known ligands, which interact with PD-1 on T cells and prevent T-cell activation and proliferation. PD-L1 is expressed on macrophages and it can be upregulated in some tissues and tumors in answer to IFN-γ and other inflammatory factors (Dong et al., 2002; Yamazaki et al., 2002; Taube et al., 2012). While, PD-L2 is expressed on macrophages and dendritic cells (Tseng et al., 2001; Ishida et al., 2002). Besides PD-1, PD-L1 can combine with CD80/B7-1 (Butte et al., 2007; Park et al., 2010) and PD-L2 can incorporate with RGMb (Xiao et al., 2014); these may cause the differences in response and immune-related adverse events (AEs) between anti-PD-1 and anti-PD-L1 antibodies.

Combination chemotherapy can cure most patients with classic Hodgkin lymphomas (cHL). However, for patients who failed to treatment (refractory cHL) or regained the disease soon (relapsed cHL), immunotherapy can be an appropriate option. CHL's typical feature is the existence of the malignant Hodgkin Reed Sternberg (HRS) cells surrounded by an inflammatory immune infiltrate. Meanwhile, PD-L1 expression was upregulated in cHL via JAK2-STAT signaling with near universal genetic amplification of the 9p24.1 locus (Green et al., 2010). Among the non-Hodgkin lymphomas (NHL), the overexpression of PD-L1 is also identified in many cases (Chen et al., 2013). Therefore, the anti-PD-1 antibody can be a potential therapy for patients with lymphoma.

The US Food and Drug Administration (FDA) currently approved two anti-PD-1 antibodies, including pembrolizumab and nivolumab. Pembrolizumab is a fully humanized IgG4 kappa isotype anti-PD-1 monoclonal antibody. Nivolumab is a fully human IgG4 anti-PD-1 monoclonal antibody. Clinic trials with other anti-PD-1 antibodies and anti-PD-L1 antibodies are ongoing, the results have not been publicated.

In recent years, immunotherapy with PD-1 blockage or PD-L1 blockage were successfully used in many cancers, including melanoma, non-small cell lung cancer, renal cell carcinoma, ovarian cancer, lymphoma, et al. (Sunshine and Taube, 2015). However, the efficacy of anti-PD-1 in lymphoma ranged widely. Additionally, the adverse events (AEs) with checkpoint inhibition is not related to traditional therapy, such as nausea, vomiting, hair loss, etc., but relates to several autoimmune side effects. However, there is no systematic review to evaluate the safety and efficacy of anti-PD-1 antibody in treating lymphoma. Therefore, this meta-analysis was to assess the safety and efficacy of anti-PD-1 antibody in patients with lymphoma, offering evidence-based references for clinicians.

## Methods

### Literature Search

We obeyed the Preferred Reporting Items for Systematic reviews and Meta-Analyses (PRISMA) guidelines. We searched PUBMED, EMBASE, and The Cochrane Library to identify the relevant studies up to March 2018. We used a combination of terms: “pembrolizumab/ lambrolizumab/ Keytruda/ MK-3475” OR “Nivolumab/ MDX-1106/ ONO-4538/ BMS-936558/ Opdivo” AND “lymphoma.”

### Inclusion and Exclusion Criteria

Studies had to meet the following criteria: (1) prospective trials concerning the efficacy or safety of nivolumab/pembrolizumab on patients with relapsed or refractory lymphoma. (2) articles reporting any of the data: ORR, 6-month PFS rate, 6-month OS rate, and drug-related AEs.

Exclusion criteria: (1) articles not association with our topics; (2) studies without usable data; and (3) retrospective or observed studies, letters, editorials, case reports, and reviews.

### Data Extraction and Quality Control

The eligible studies were reviewed and extracted data by two authors independently. We extracted first author, published year, ClinicalTrials.gov number, phase, study design, treatment, disease, number of patients, age, prior systemic treatment regimens, ORR, 6-month PFS rate, 6-month OS rate, any grade AEs, grader ≥3 AEs, and drug-related deaths. The methodological index for non-randomized studies (MINORS) (Slim et al., 2015) was used to evaluate the methodological quality of the included articles. MINORS contained 12 items, the first eight being specifically for non-comparative studies. The items including a stated aim of the study, the inclusion of consecutive patients, prospective collection of data, endpoint appropriate to the study aim, unbiased evaluation of endpoints, follow-up period appropriate to the major endpoint, loss to follow up not exceeding 5% and prospective calculation of the sample size. Each item was scored from 0 to 2; 0 indicates that it was not reported, one represented that it was reported inadequately, and 2 revealed that it was reported adequately.

### Statistical Analysis

The primary outcome for efficacy was ORR; secondary outcomes were 6-month PFS and 6-month OS. For safety analysis, the incidence and exhibition of any grade and grade ≥3 AEs were evaluated. In each trial, objective response rate (ORR) = [(complete responses + partial responses) ÷ total no. of patients] × 100. Heterogeneity among studies was detected with a forest plot and the inconsistency statistic (I^2^). A random-effect model was used when potential heterogeneity existed (I^2^ >50%); otherwise, the fixed-effect model was employed. The Metaprop module in the R-3.3.2 statistical software package was used to analyze the efficacy and safety. Subgroup analysis was performed to solve heterogeneity. Sensitivity analysis was carried out by using different effect models. No dose effect was considered. *P* < 0.05 suggested statistically significant.

## Results

### Study Selection

**Figure 1:**
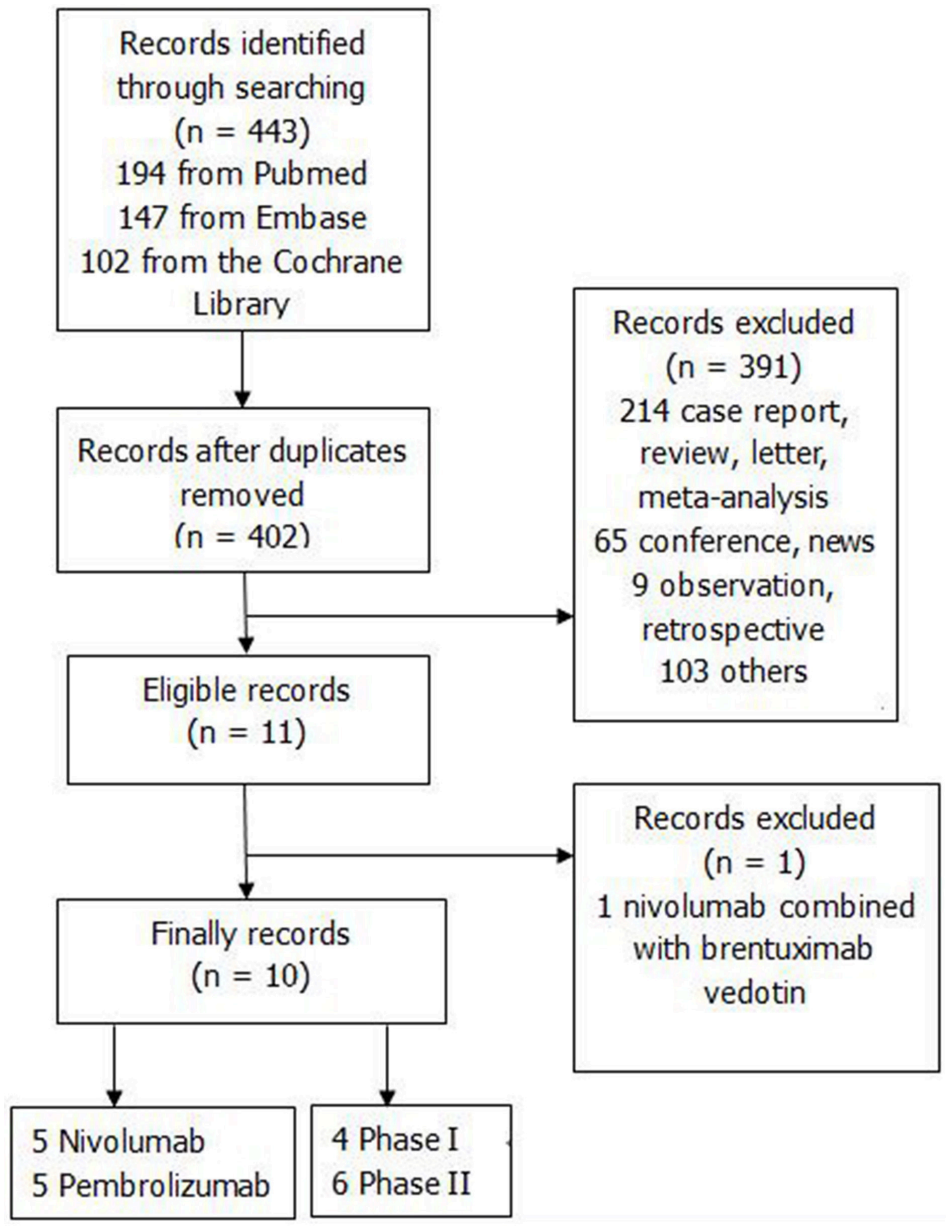
The flow chart.

The search strategy produced a total of 443 records; 41 studies were removed after duplication; 391 studies were excluded. Finally, ten studies were enrolled after removing one study with combined therapy (Ansell et al., 2015; Armand et al., 2016, 2018; Lesokhin et al., 2016; Younes et al., 2016; Chen et al., 2017; Ding et al., 2017; Maruyama et al., 2017; Zinzani P. et al., 2017; Zinzani P. L. et al., 2017). Figure 1 showed the procedure of study selection.

### Study Characteristics

Table 1 showed the characteristics of the included studies. The included studies were published from 2015 to 2018. We included a total of 10 studies, 762 patients, of which 114 patients were NHL [9 CLL with Richter transformation, 105 primary mediastinal large B-cell lymphomas (PMBCL)], 604 patients were HL, 17 patients were leukemia, and 27 patients were multiple myeloma. Altogether 384 patients' mean ages were < 50 years, while 79 patients' mean ages were >50 years. We assessed AEs, ORR, PFS and OS only in patients with lymphoma. There were 4 phase I studies and 6 phase II studies. Patients in 5 studies used nivolumab and five studies received pembrolizumab. Two studies were dose-escalation, cohort expansion studies, three studies were multicohort studies, and five studies were single-arm trails. Patients received nivolumab intravenously at a dose of 1 or 3 mg/kg every 2 weeks. Pembrolizumab were given 10 mg/kg every 2 weeks or 200 mg every 3 weeks. Drug-related deaths occurred in two patients; one received nivolumab with pneumonitis/ARDS, one treated with pembrolizumab observed with Pseudomonas sepsis.

**Table 1:** The characteristics of included studies.

**No**.	**Study**	**Clinic trials gov**.	**Phase**	**Study design**	**Treatment**	**Disease**	**No. of patients**	**Ages(years), medium (range)**	**Median prior systemic treatment regimens**	**Drug-related adverse events(any grade, n)**	**Drug-related adverse events(grade≥3, n)**	**Drug-related deaths(n)**	**ORR(%)**	**6-month PFS rate(%)**	**6-month OS rate(%)**
**NIVOLUMAB**															
1	Lesokhin et al. (2016)	NCT01592370	I	dose-escalation, cohort-expansion	nivolumab, 1 or 3 mg/kg every 2 weeks	relapsed or refractory NHL/MM	82:27 MM;31 B-NHL; 23 T-NHL; 1 CML	MM: 63(32–81) B-NHL: 65(23–74) T-NHL:61(30–81)	3(1–12)	51	18	1(pneumonitis/ARDS)	3.70%; 22.22%; 0	–	–
2	Ansell et al. (2015)	NCT01592370	I	dose-escalation, cohort-expansion	nivolumab, 1 or 3 mg/kg every 2 weeks	relapsed or refractory HL	23	35(20–54)	–	18	5	0	87%	86%	–
3	Maruyama et al. (2017)	JapicCTI-142755	II	multicenter, single-arm	nivolumab, 3 mg/kg every 2 weeks	relapsed or refractory HL	17	63(29–83)	3 (2–5)	17	4	0	75%	60%	100%
4	Younes et al. (2016)	NCT02181738	II	multicentre, multicohort, single-arm	nivolumab, 3 mg/kg every 2 weeks	relapsed or refractory HL	80	37 (28–48)	4 (4–7)	71	20	0	66%	77%	99%
5	Armand et al. (2018)	CheckMate 205	II	multicohort, single-arm	nivolumab 3 mg/kg every 2 weeks	relapsed/refractory cHL	243	–	–	–	–	0	69%	–	–
**PEMBROLIZUMAB**															
1	Armand et al. (2016)	NCT01953692	Ib	multicohort	pembrolizumab, 10 mg/kg every 2 weeks	relapsed or refractory HL	31	32(20–67)	–	21	5	0	65%	69%	100%
2	Chen et al. (2017)	NCT02453594	II	multicenter single-arm	pembrolizumab, 200 mg once every 3 weeks	relapsed/refractory HL	210	35(18–76)	4(1–12)	–	–	0	69%	72.40%	99.50%
3	Ding et al. (2017)	NCT02332980	II	single-arm	pembrolizumab, 200 mg every 3 weeks	relapsed and transformed CLL	25: 16 relapsed or refractory CLL; 9 RT	69(46–81)	4(1–10)	25	15	1(Pseudomonas sepsis)	0; 44%	–	57.14%; 71.43%
4	Zinzani et al. (2018)	NCT01953692	Ib	multicenter, international, multicohort	pembrolizumab, 10 mg/kg every 2 weeks(10); 200 mg every 3 weeks(8)	relapsed/refractory primary mediastinal large B-cell lymphoma (rrPMBCL)	18	30(22–62)	4(2–6)	11	2	0	41%	–	–
5	Zinzani P. L. et al. (2017)	NCT02576990	II	two -cohort, multicenter	pembrolizumab 200 mg IV every 3 weeks	relapsed/refractory primary mediastinal large B-cell lymphoma (rrPMBCL)	33	32(20–58)	3(1–5)	19	6	0	35%	–	–

*CTC for AE version, Common Terminology Criteria for Adverse Events version; n/a, non-available. HL, Hodgkin's lymphoma; NHL, non-Hodgkin's lymphoma; MM, multiple myeloma; T-NHL, T cells non-Hodgkin's lymphoma; B-NHL, B cells non-Hodgkin's lymphoma; CML, chronic myeloid leukemia; CLL, chronic lymphocytic leukemia; RT, Richter transformation*.

### Safety

**Figure 2:**
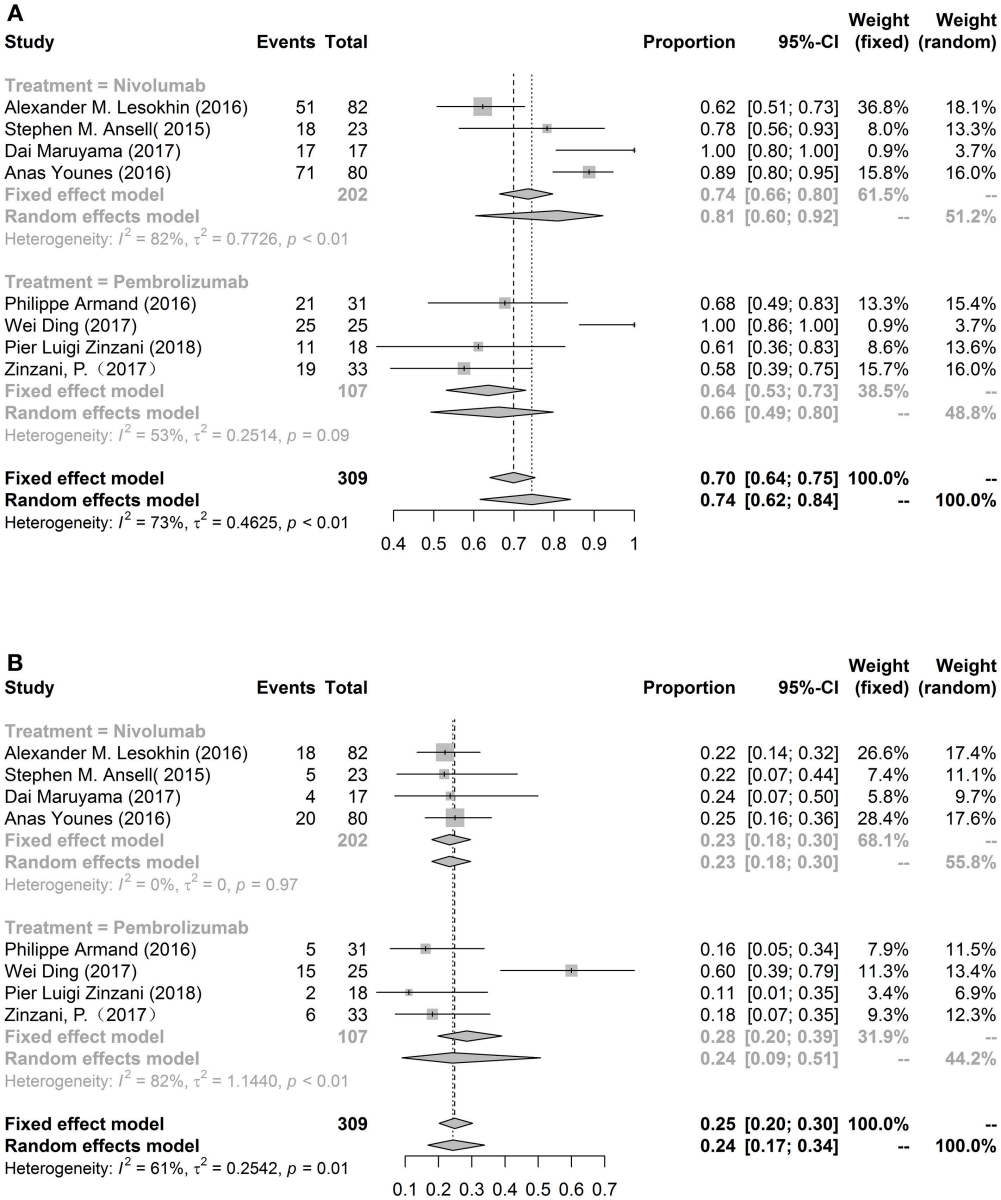
The forest plot of pooled incidence of AEs in any grade **(A)** and grade ≥3 **(B)**.

Overall eight studies were included to assess the pooled incidence of any grade (74%, 95%CL: 62%−84%) and grade ≥3 (24%, 95%CL: 17%−34%) AEs (Figure 2). There was no significant difference in the total risk of AEs between the nivolumab and pembrolizumab. The most common any grade adverse event was fatigue (14.91%, 10.27%−21.13%). Other common drug-related any grade AEs were rash (14.8%), hypothyroidism (13.77%), platelet count decreased (13.54%), pyrexia (13%), cough (11.56%), pruritus (10.81%), and nausea (10.16%). Neutropenia was the most common grade ≥3 AEs (4.79%). Another common severe AEs were pneumonitis (3.58%), rash (3.38%), and leukopenia (3.31%). We also compared nivolumab with pembrolizumab in patients with lymphoma, the incidences of any grade fatigue (*p* = 0.0072) and rash (*p* = 0.0078) were lower in pembrolizumab group than those patients with nivolumab. More details were exhibited in Table 2.

**Table 2:** The incidence of adverse events in all grade or grade ≥3.

		**Any grade**						**Grade ≥3**					
**AEs**	**Treatment**	**Included study**	**Event**	**Total patients**	**Pooled rate (95%Cl)**	**Model**	***P*-value**	**Included study**	**Event**	**Total patients**	**Pooled rate (95%Cl)**	**Model**	***P*-value**
**GENERAL**													
Fatigue	Nivolumab	4	40	202	0.2018 [0.1514; 0.2636]	Fixed	**0.007**	4	0	202	0.0121 [0.0030; 0.0471]	Fixed	0.2200
	Pembrolizumab	4	28	284	0.1024 [0.0715; 0.1445]	Fixed		3	3	253	0.0280 [0.0044; 0.1595]	Random	
	Overall	8	68	486	0.1491 [0.1027; 0.2113]	Random		7	3	455	0.0245 [0.0113; 0.0524]	Fixed	
Pyrexia	Nivolumab	3	16	122	0.1675 [0.0486; 0.4422]	Random	0.442	3	1	122	0.0367 [0.0105; 0.1199]	Fixed	0.49
	Pembrolizumab	3	26	253	0.1030 [0.0711; 0.1471]	Fixed		3	2	253	0.0208 [0.0073; 0.0579]	Fixed	
	Overall	6	42	375	0.1300 [0.0728; 0.2213]	Random		6	3	375	0.0263 [0.0118; 0.0575]	Fixed	
Chills	Pembrolizumab	3	9	266	0.0386 [0.0202; 0.0727]	Fixed		2	0	235	0.0067 [0.0009; 0.0463]	Fixed	
Asthenia	Pembrolizumab	2	5	241	0.0288 [0.0064; 0.1199]	Random		1	1	33	0.0303		
**RESPIRATORY**													
Pneumonitis	Nivolumab	2	12	162	0.0629 [0.0129; 0.2571]	Random	0.859	2	5	162	0.0370 [0.0155; 0.0860]	Fixed	0.853
	Pembrolizumab	2	4	49	0.0840 [0.0319; 0.2037]	Fixed		1	1	33	0.0303		
	Overall	4	15	211	0.0942 [0.0574; 0.1508]	Fixed		3	6	195	0.0358 [0.0161; 0.0775]	Fixed	
Embolism	Nivolumab	2	1	162	0.0138 [0.0035; 0.0535]	Fixed		2	1	162	0.0138 [0.0035; 0.0535]	Fixed	
Cough	Nivolumab	1	2	23	0.0879			1	0	23	0		
	Pembrolizumab	2	19	235	0.1297 [0.0236; 0.4793]	Random		2	1	235	0.0091 [0.0023; 0.0357]	Fixed	
	Overall	3	21	258	0.1156 [0.0346; 0.3226]	Random		3	1	258	0.0107 [0.0031; 0.0364]	Fixed	
Upper respiratory tract infection	Nivolumab	2	5	97	0.0603 [0.0010; 0.8038]	Random		2	0	97	0.0130 [0.0018; 0.0871]	Fixed	
	Pembrolizumab	1	13	210	0.0619			1	0	210	0		
	Overall	3	18	307	0.0780 [0.0147; 0.3243]	Random		3	0	307	0.0074 [0.0015; 0.0358]	Fixed	
Dyspnea	Nivolumab	1	3	80	0.0375			1	1	80	0.0125		
	Pembrolizumab	3	17	266	0.0951 [0.0172; 0.3873]	Random		2	6	235	0.0424 [0.0024; 0.4524]	Random	
	Overall	4	20	346	0.0764 [0.0202; 0.2489]	Random		3	7	315	0.0298 [0.0036; 0.2068]	Random	
**SKIN**													
Rash	Nivolumab	4	35	202	0.1927 [0.1146; 0.3057]	Random	**0.008**	4	5	202	0.0366 [0.0175; 0.0749]	Fixed	0.651
	Pembrolizumab	2	18	235	0.0766 [0.0488; 0.1183]	Fixed		2	1	235	0.0145 [0.0006; 0.2577]	Random	
	Overall	6	53	437	0.1480 [0.0869; 0.2408]	Random		6	6	437	0.0338 [0.0176; 0.0639]	Fixed	
Pruritus	Nivolumab	4	24	202	0.1403 [0.0707; 0.2593]	Random		4	0	202	0.0121 [0.0030; 0.0471]	Fixed	
	Pembrolizumab	1	8	210	0.0381			1	0	210	0		
	Overall	5	32	412	0.1081 [0.0509; 0.2152]	Random		5	0	412	0.0087 [0.0025; 0.0296]	Fixed	
**GASTROINTESTINAL**													
Decreased appetite	Nivolumab	2	9	162	0.0532 [0.0160; 0.1621]	Random	0.645	2	0	162	0.0061 [0.0009; 0.0420]	Fixed	
	Pembrolizumab	2	4	49	0.0844 [0.0320; 0.2045]	Fixed		1	0	18	0		
	Overall	4	13	211	0.0701 [0.0410; 0.1172]	Fixed		3	0	180	0.0099 [0.0020; 0.0476]	Fixed	
Diarrhea	Nivolumab	4	19	202	0.0959 [0.0620; 0.1455]	Fixed	0.9710	4	0	202	0.0121 [0.0030; 0.0471]	Fixed	0.853
	Pembrolizumab	4	26	284	0.0969 [0.0667; 0.1387]	Fixed		3	2	253	0.0142 [0.0050; 0.0398]	Fixed	
	Overall	8	45	486	0.0965 [0.0727; 0.1269]	Fixed		7	2	455	0.0134 [0.0058; 0.0306]	Fixed	
Nausea	Nivolumab	2	13	103	0.1262 [0.0747; 0.2054]	Fixed	0.305	2	0	103	0.0113 [0.0016; 0.0761]	Fixed	0.958
	Pembrolizumab	4	23	284	0.1069 [0.0547; 0.1983]	Random		3	0	253	0.0106 [0.0021; 0.0509]	Fixed	
	Overall	6	36	387	0.1016 [0.0741; 0.1380]	Fixed		5	0	356	0.0109 [0.0031; 0.0369]	Fixed	
Vomiting	Nivolumab	1	6	80	0.0750			1	0	80	0		
	Pembrolizumab	3	13	266	0.0533 [0.0311; 0.0899]	Fixed		2	0	235	0.0067 [0.0009; 0.0463]	Fixed	
	Overall	4	19	346	0.0594 [0.0381; 0.0913]	Fixed		3	0	315	0.0065 [0.0013; 0.0318]	Fixed	
Constipation	Nivolumab	2	7	97	0.0746 [0.0359; 0.1484]	Fixed	0.134	2	0	97	0.0130 [0.0018; 0.0871]	Fixed	
	Pembrolizumab	2	8	241	0.0349 [0.0175; 0.0683]	Fixed		1	0	210	0		
	Overall	4	15	338	0.0495 [0.0300; 0.0807]	Fixed		3	0	307	0.0074 [0.0015; 0.0358]	Fixed	
Stomatitis	Nivolumab	2	4	105	0.0456 [0.0171; 0.1157]	Fixed		2	2	105	0.0229 [0.0057; 0.0873]	Fixed	
**HEPATIC**													
Lipase increased	Nivolumab	2	5	105	0.0514 [0.0215; 0.1179]	Fixed		4	18	428	0.0454 [0.0288; 0.0710]	Fixed	
AST increased	Nivolumab	1	4	80	0.0500			1	2	80	0.0250		
	Pembrolizumab	1	2	31	0.0645			1	1	31	0.0323		
	Overall	2	6	111	0.0544 [0.0246; 0.1159]	Fixed		2	3	111	0.0272 [0.0088; 0.0810]	Fixed	
ALT increased	Nivolumab	1	3	80	0.0375			2	9	323	0.0279 [0.0146; 0.0528]	Fixed	
	Pembrolizumab	1	2	31	0.0645			1	1	31	0.0323		
	Overall	2	5	111	0.0465 [0.0195; 0.1070]	Fixed		3	10	354	0.0283 [0.0153; 0.0518]	Fixed	
**ENDOCRINE**													
Hypothyroidism	Nivolumab	2	7	40	0.1789 [0.0493; 0.4780]	Random	0.251	2	0	40	0.0241 [0.0034; 0.1520]	Fixed	0.463
	Pembrolizumab	3	33	259	0.1279 [0.0924; 0.1745]	Fixed		2	1	228	0.0098 [0.0025; 0.0385]	Fixed	
	Overall	5	40	299	0.1377 [0.1025; 0.1826]	Fixed		4	1	268	0.0132 [0.0043; 0.0403]	Fixed	
**NEURAL**													
Headache	Nivolumab	2	6	97	0.0844 [0.0080; 0.5126]	Random		2	0	97	0.0130 [0.0018; 0.0871]	Fixed	
	Pembrolizumab	1	13	210	0.0619			1	0	210	0		
	Overall	3	19	307	0.0768 [0.0246; 0.2155]	Random		3	0	307	0.0074 [0.0015; 0.0358]	Fixed	
Back pain	Nivolumab	2	4	97	0.0544 [0.0113; 0.2244]	Random		2	0	97	0.0130 [0.0018; 0.0871]	Fixed	0.669
	Pembrolizumab	1	4	210	0.0190			2	1	241	0.0132 [0.0007; 0.2022]	Random	
	Overall	3	8	307	0.0358 [0.0119; 0.1026]	Random		4	1	338	0.0184 [0.0059; 0.0559]	Fixed	
Myalgia	Nivolumab	2	8	97	0.0838 [0.0424; 0.1587]	Fixed		2	0	97	0.0130 [0.0018; 0.0871]	Fixed	
	Pembrolizumab	1	5	210	0.0238			1	0	210	0		
	Overall	3	13	307	0.0552 [0.0216; 0.1343]	Random		3	0	307	0.0074 [0.0015; 0.0358]	Fixed	
Arthralgia	Nivolumab	1	11	80	0.1375			1	0	80	0.0062		
	Pembrolizumab	1	8	210	0.0381			1	1	210	0.0048		
	Overall	2	19	290	0.0742 [0.0201; 0.2388]	Random		2	1	290	0.0069 [0.0017; 0.0270]	Fixed	
**HEMATOLOGIC**													
Anemia	Nivolumab	2	8	162	0.0507 [0.0256; 0.0982]	Fixed	0.955	2	3	162	0.0331 [0.0124; 0.0850]	Fixed	0.945
	Pembrolizumab	2	11	235	0.0579 [0.0005; 0.8856]	Random		2	5	235	0.0296 [0.0003; 0.7551]	Random	
	Overall	3	16	317	0.0576 [0.0098; 0.2741]	Random		3	8	317	0.0292 [0.0046; 0.1631]	Random	
Neutropenia	Nivolumab	2	10	162	0.0670 [0.0364; 0.1203]	Fixed	0.649	3	12	405	0.0322 [0.0183; 0.0558]	Fixed	0.243
	Pembrolizumab	3	14	253	0.0558 [0.0333; 0.0921]	Fixed		4	14	286	0.0685 [0.0216; 0.1969]	Random	
	Overall	5	24	415	0.0602 [0.0407; 0.0883]	Fixed		7	26	691	0.0479 [0.0234; 0.0953]	Random	
Lymphopenia	Nivolumab	3	8	185	0.0474 [0.0239; 0.0921]	Fixed		3	5	185	0.0276 [0.0115; 0.0647]	Fixed	
Platelet count decreased	Nivolumab	2	5	103	0.0553 [0.0038; 0.4766]	Random		2	0	103	0.0113 [0.0016; 0.0761]	Fixed	
	Pembrolizumab	1	11	25	0.4400			1	5	25	0.2000		
	Overall	3	16	128	0.1354 [0.0225; 0.5159]	Random		3	5	128	0.0414 [0.0034; 0.3530]	Random	
Leukopenia	Nivolumab	2	6	162	0.0390 [0.0176; 0.0841]	Fixed		2	3	162	0.0331 [0.0124; 0.0850]	Fixed	

*Bold values indicates the classification of the adverse events*.

### Efficacy

**Figure 3:**
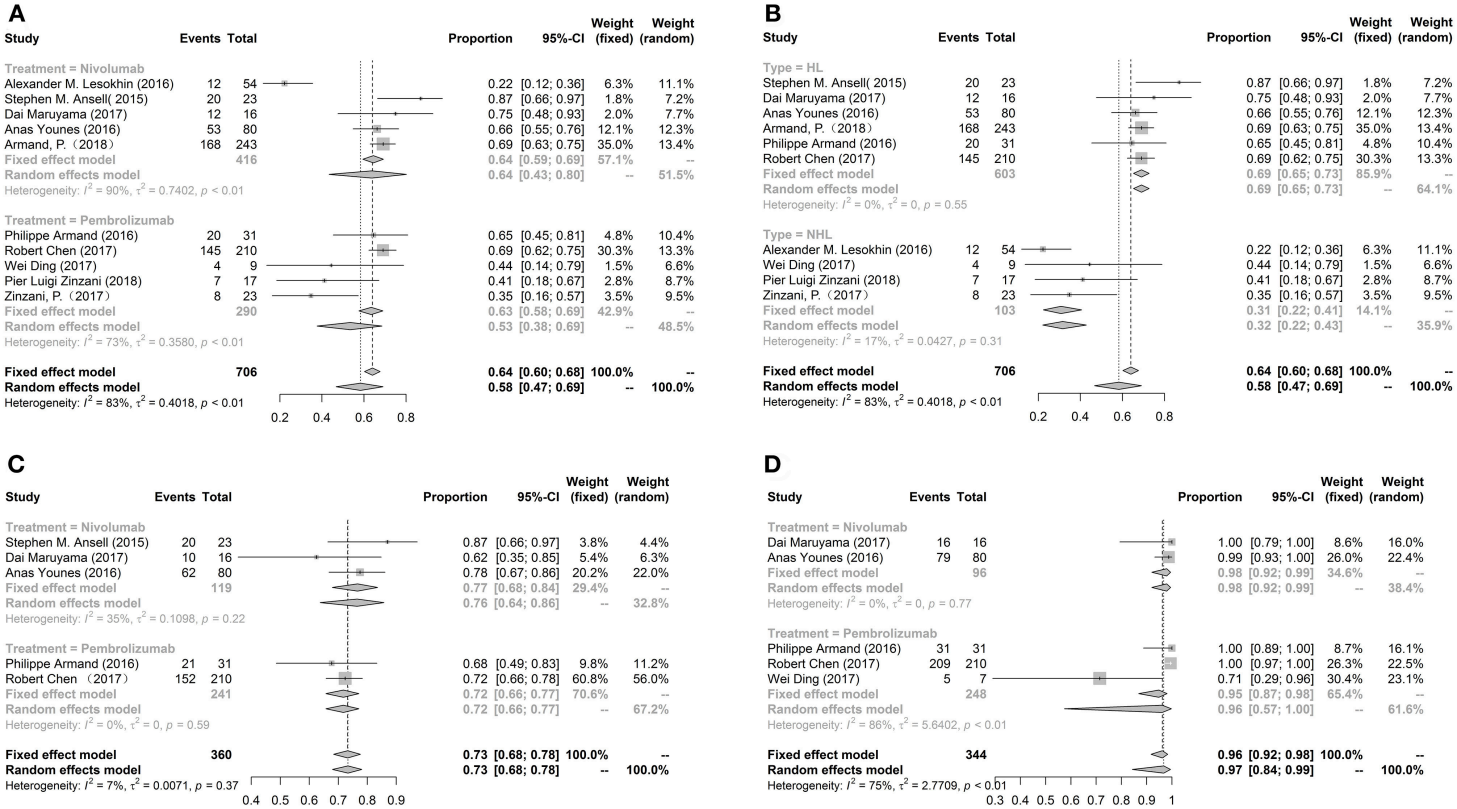
The forest plot of pooled ORR **(A)**, 6-month PFS rate **(C)**, 6-month OS rate **(D)** in patients received nivolumab or pembrolizumab; the forest plot of pooled ORR in patients with HL or NHL **(B)**.

The pooled ORR, 6-month PFS rate and 6-month OS rate were performed to evaluate the efficacy of nivolumab or pembrolizumab treated lymphoma. We enrolled all ten studies to analyze ORR, five studies to evaluate PFS and five studies to assess OS. The pooled ORR, PFS rate and OS rate were 58% (95%Cl: 47%−69%), 73% (95%Cl: 68%−78%), and 96% (95%Cl: 92%−98%), respectively. There were no significant differences in ORR between patients' mean age >50 years (46%, 16%−79%) and < 50 years (62%, 50%−73%). PFS and OS between patients' mean age >50 and < 50 years did not analyze due to limitation numbers. Meanwhile, the ORR, PFS and OS between nivolumab and pembrolizumab had no significant differences. While, the ORR in patients with HL was higher than patients with NHL (69.08 vs. 30.77%, *p* < 0.0001). The PFS and OS could not be subgrouped by HL and NHL. These results were exhibited in Figure 3.

### Study Quality

Two studies without full text can't evaluate totally. The two items including unbiased evaluation of endpoints and prospective calculation of the sample size were not reported. The overall score was high. Therefore, the overall quality of the included studies was satisfactory (Table 3).

**Table 3:** The scores of MINORS.

**References**	**1**	**2**	**3**	**4**	**5**	**6**	**7**	**8**	**Total**
Lesokhin et al. (2016)	2	2	2	2	0	2	2	0	12
Ansell et al. (2015)	2	2	2	2	0	2	2	0	12
Maruyama et al. (2017)	2	2	2	1	0	2	2	0	11
Younes et al. (2016)	2	2	2	2	0	2	2	0	12
Armand et al. (2018)	2	–	–	2	–	2	–	–	6
Armand et al. (2016)	2	2	2	2	0	2	2	0	12
Chen et al. (2017)	2	2	2	2	0	2	2	0	12
Ding et al. (2017)	2	2	2	1	0	2	2	0	11
Zinzani et al. (2018)	2	2	2	1	0	2	2	0	11
Zinzani P. L. et al. (2017)	2	–	–	2	–	–	–	–	4

## Discussion

Anti-PD-1 antibodies are rapidly developed in recent decades. FDA has approved two anti-PD-1 antibodies, including pembrolizumab and nivolumab. However, the AEs may be different between pembrolizumab and nivolumab. Meanwhile, the efficacy of these two anti-PD-1 antibodies in lymphoma ranged widely.

This meta-analysis included overall ten prospective studies with 718 patients with lymphomas, including 114 patients with NHL and 604 patients with HL, to assess the safety and efficacy. The pooled incidence of AEs of any grade reached 74%, while grade ≥3 was only 24%. However, there were two patients occurred drug-related death. Approximately 58% of patients gained complete response or partial response. Meanwhile, 73% of patients' diseases remained stable for half a year, and 96% of patients survived for half a year.

Immune-related adverse events caused by blockage of the PD-1 pathway can affect almost any organ, mainly mediated by T cells (Weber et al., 2015b). B cells secreting antibodies (Good-Jacobson et al., 2016) and granulocytes secreting inflammatory mediators and cytokines (Zitvogel and Kroemer, 2012; Good-Jacobson et al., 2016) may also develop immune-related adverse events. We found that the most common any grade adverse event were fatigue, rash, hypothyroidism, platelet count decreased, pyrexia, cough, pruritus, and nausea. The severe AEs over 3% were neutropenia, pneumonitis, rash, and leukopenia. In advanced melanoma, fatigue (19–21%, 34%), diarrhea (14–17%, 11–19%), pruritus (14%, 16–19%), rash (13–15%, 9–22%), arthralgia (9–12%, 6–8%), vitiligo (9–11%, 5–11%) and hypothyroidism (9–10%, 4–9%) were most common for any grade in pembrolizumab (Robert et al., 2015b) and nivolumab (Larkin et al., 2015; Robert et al., 2015a; Weber et al., 2015a), respectively. In advanced lung cancer, fatigue (14%, 16%), diarrhea (8%, 8–10%), pruritus (11%, 6–8%), rash (10%, 4–11%), arthralgia (9%, 5%), hypothyroidism (8%, 4–7%) and pneumonitis (8%, 4–7%) were most common for any grade in pembrolizumab (Garon et al., 2015; Herbst et al., 2016) and nivolumab (Borghaei et al., 2015; Brahmer et al., 2015; Rizvi et al., 2015), respectively. Therefore, the safety of anti-PD-1 antibodies were similar between the different cancers.

Many clinic trials reported fatigue as one of the AEs with anti-PD-1 antibodies (Brahmer et al., 2015; Rizvi et al., 2015). While, it was generally mild and not related to other systemic symptoms. We reported the maculopapular rash was most commonly. Additionally, rarer rashes including lichenoid (Joseph et al., 2015), bullous pemphigoid (Carlos et al., 2015), Stevens-Johnson syndrome, and toxic epidermal necrolysis (Postow, 2015) were also described and may be life-threatening. Immune-modulating medications like corticosteroids were usually utilized to treat the rash. Pyrexia was described in multiple immunotherapy, including cancer vaccines, adoptive T-cell therapy, chimeric antigen receptor T cells, and antibodies (Weber et al., 2015b). The cytokine release and nonspecific activation of an immune response may cause this AEs (Schwartz et al., 2002). Antipyretics such as acetaminophen or nonsteroidal anti-inflammatory drugs may solve the problem. Hypothyroidism was another common AEs, which can be managed with thyroid hormone replacement. Pneumonitis was common both in any grade and severe AEs. If pneumonitis grade >1, infectious diseases physicians and pulmonologist should exclude infectious etiologies, and oral or intravenous corticosteroids may be needed. Diarrhea and nausea are most commonly AEs in gastrointestinal disorders. Mild diarrhea can be cured with diet and antidiarrheal medications including atropine and oral diphenoxylate hydrochloride (Postow, 2015). Worsening or persistent diarrhea for more than 3 days should consider an infectious cause. Therefore, early detect and properly manage these immune-related AEs are very important. Additionally, the trails compared AEs of anti-PD-1 and traditional therapy should be performed to find an optimal treatment.

Arthritis, myositis, sicca syndrome, vasculitis were common AEs for anti-PD-1 antibodies in the type of rheumatology. Several studies suggested that patients with underlying autoimmunity, including rheumatic diseases, can be effectively treated by immune checkpoint inhibitors, but 1/3 of patients may occur the outbreak of underlying diseases (Johnson et al., 2016; Maul et al., 2016; Menzies et al., 2017). Therefore, rheumatologists and oncologists were needed to care of such patients and to explore the potential mechanisms of these complications (Calabrese and Mariette, 2018).

Former study (Lee et al., 2016) found that nivolumab and pembrolizumab combine with similar areas, but another study (Tan et al., 2017) suggested that the two antibodies bind to completely different areas of PD-1. The pembrolizumab mainly binds to the C'D loop of PD-1, while nivolumab primarily binds to the N-loop, which is not involved in recognition of PD-L1. We found no difference in ORR, PFS and OS. However, the incidences of any grade AEs like fatigue and rash were lower in pembrolizumab than nivolumab, consistent with the previous study with lymphoma (Xu-Monette et al., 2017) and with advanced melanoma (Spain et al., 2016). This difference may because the different structures which the anti-PD-1 agents bind to play a different role in downstream cytokine signaling. Therefore, more randomized controlled trials are needed to detect the difference of safety between two agents, and further basic experiments are needed to explore the potential mechanism.

Generally, the expression of PD-1 is usually elevated on tumor-infiltrating T cells (TILs) in lymphomas, especially observed in HL (Yamamoto et al., 2008; Muenst et al., 2009) than in NHL (Ahearne et al., 2014; Kiyasu et al., 2015; Kwon et al., 2016). Similarly, we showed that the ORR in patients with HL was higher than NHL. It may suggest that the anti-tumor activity is an association with PD-1 expression. Additionally, PD-L1/PD-L2 expression often increased in cHL (97%) (Roemer et al., 2016) and PMBCL (70%) (Green et al., 2010) because of copy-number gain or amplification of 9p24.1. Meanwhile, Epstein-Barr virus (EBV) infection also may lead PD-L1 overexpression in HL (Kieser et al., 1997; Green et al., 2012; Ok et al., 2013). Therefore, anti-PD-1 antibodies inhibited PD-L1/PD-L2 binding to PD-1, increasing the anti-tumor activity of T cells in HL. We could not evaluate the differences of PFS and OS between HL and NHL due to the limitation of study number. However, some studies showed that high expression of PD-1 on TILs was related to poor prognosis (OS) (Muenst et al., 2009) and disease-specific survival (Greaves et al., 2013). Therefore, more randomized controlled trials are needed to detect the difference of efficacy between HL and NHL.

Previous study (Georgieva et al., 2018) has demonstrated that first-line pembrolizumab for non-small cell lung cancer may be cost-effective in the US but not the UK, in spite of very similar incremental cost-effectiveness ratios values in both countries. Therefore, the cost must be considered to use anti-PD-1 antibodies for patients.

Our study has several limitations. First, the study number was limited, which may make the data skewed. Second, there were only phase I/II studies without double-blinded RCT, which may lead the potential performance bias. Third, the survival time and PFS time didn't present individually, so we can't perform the survival analysis.

In conclusion, this meta-analysis demonstrated that nivolumab and pembrolizumab have potential effects of ORR, 6-month OS rate and 6-month PFS rate, while the AEs and drug-related deaths were tolerable in patients with relapsed or refractory lymphoma. We also demonstrated that pembrolizumab had a lower risk of AEs than nivolumab, and patients with HL had a better ORR than NHL. Further researches with these novel drugs are needed to compare with traditional therapy for patients with relapsed or refractory lymphoma.

## Author Contributions

HZ collected, analyzed the data and wrote the article. XF and QL collected data, prepared the pictures and tables. TN provided the idea and modified the article. All authors read and approved the final manuscript.

### Conflict of Interest Statement

The authors declare that the research was conducted in the absence of any commercial or financial relationships that could be construed as a potential conflict of interest.
